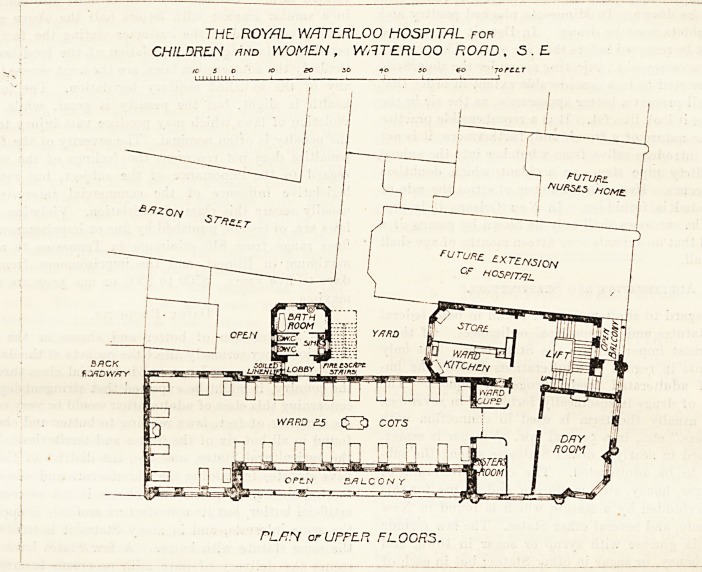# The Royal Waterloo Hospital for Women and Children, Waterloo Road, S.E.

**Published:** 1906-02-03

**Authors:** 


### HOSPITAL ADMINISTRATION.

### CONSTRUCTION AND ECONOMICS.

### THE ROYAL WATERLOO HOSPITAL FOR WOMEN AND CHILDREN, WATERLOO ROAD, S.E.

At the corner of Stamford Street and Waterloo Road is the entrance to the upper ground floor. Here there is a fine entrance hall from which are reached the main staircase with its lift, the porter's room, secretary's office, and a corridor which gives access to the board-room and medical officers' rooms.

The first, second, and third floors contain the new wards. Each ward has attached to it a nurses' room, a store-room, a ward kitchen, and of course the staircase and lifts. Near the centre of one side of the ward is the sanitary annexe containing the closets and bath-room. This annexe is properly separated from the ward by a cross-ventilated passage, and it has attached to it a fire-escape staircase. A large balcony is incorporated with the side of the ward which overlooks Waterloo Road, and it is a striking and valuable feature in the construction. The first-floor ward contains 25 beds, and there is a good-sized day-room at the opposite side of the passage where it is readily accessible either from the main staircase, the ward, or the sisters' room. The second and the third floors are similar to the first floor except that the space above the day-room is used on the second floor as an isolation ward, and on the third floor as an operating theatre. The fourth floor is occupied by the kitchen offices and by bedrooms for the staff. The floors are of fireproof construction throughout, and fire-hydrants will be placed on each floor. The heating will be by open fireplaces and hot-water radiators.

The elevations are carried out in red brick and terra cotta, but the entrance porch is of glazed ware, and the cost of this part has been defrayed by Messrs. Doulton and Company.

GROUND FLOOR PLAN.

Considering the available space at the architect's disposal it must be said that the arrangements of the various sections of the hospital are good; and in the large wards by far the greater number of beds have windows on both sides. No doubt had more space been possible this rule would have been followed in every instance; the sanitary annexe would have been placed at the corner of the block; the kitchen would not have extended so far along the side of the ward; and the dead wall at the end would have had windows in it; but in London it is nearly impossible to plan the component parts of a hospital in the same way that it could be done on a country site; and the architect who can design a well-lighted, well-ventilated, easily workable hospital on a limited town-space is always deserving of praise. This is a case in point.

Provision is made at the Waterloo Hospital for future extension, and the proposed additions are outlined on the plan. It is clear, however, that part at least of these future extensions cannot be altogether satisfactory. For instance, the other side would be shut out from light by the Nurses' Home and the Sanitary Annexe.

The cost of the completed buildings will be £60,000. The the larger outlined space, which is probably intended for a ward, would have two-thirds of one side blocked by the wall of the main staircase and store-room; and more than half of architects are Messrs. Waring and Nicholson, of 38 Parliament Street, and the contractors are Messrs. Holliday and Greenwood, of Brixton.

## Figures and Tables

**Figure f1:**
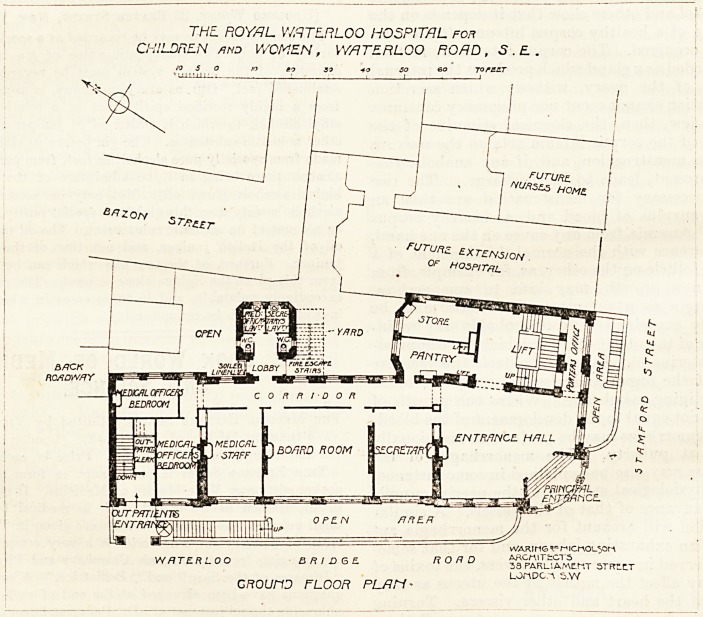


**Figure f2:**